# Bacteremia Caused by Clostridium symbiosum in University Clinical Hospital Mostar: A Case Report

**DOI:** 10.7759/cureus.71974

**Published:** 2024-10-20

**Authors:** Doris Martinovic Rizikalo, Sanja Jakovac, Tanja Petrovic, Maja Kljakic, Andrea Misetic, Ruza Coric

**Affiliations:** 1 Department of Microbiology and Molecular Diagnostics, University Clinical Hospital Mostar, Mostar, BIH; 2 School of Medicine, University of Mostar, Mostar, BIH

**Keywords:** anaerobes, bacteremia, clostridium symbiosum, maldi-tof ms, malignancy

## Abstract

*Clostridium spp*. is one of the most common anaerobic pathogens that cause bacteremia. It is often associated with intraabdominal sepsis due to trauma or surgery, and malignancies or diabetes. This is the presentation of a *Clostridium symbiosum *(*C. symbiosum*)bacteremia in a 66-year-old woman with a history of bladder cancer and radical cystectomy with ureterosigmoidostomy. The isolate was identified by matrix-assisted laser desorption/ionization time-of-flight mass spectrometry (MALDI-TOF MS, Bruker, Ettlingen, Germany). The application of new methods, such as MALDI-TOF MS and 16S rRNA gene sequencing, has made it possible to provide rapid and reliable identification of diverse anaerobic species and timely information about their role in infections.

## Introduction

Anaerobic bacteria are an important cause of many invasive infections and they can be clinically significant pathogens in bloodstream infections and septicemia, even though they have a relatively low prevalence (around 7-10% of all bacteremic episodes) [1,2]. The mortality rate associated with anaerobic bacteremia is very high, varying between 25% and 44% [3]. Factors that are frequently associated with higher mortality from anaerobic bacteremia include underlying malignancies, diabetes, and gastrointestinal surgery [4].

The majority of anaerobic bacteremia is caused by *Bacteroides fragilis* group. *Peptostreptococcus*, *Clostridium*, and *Fusobacterium *are the other species that cause anaerobic bacteremia [5]. *Clostridium* spp. cause about 0.5-2% of all significant blood culture isolates, most of which are caused by *Clostridium perfringens*. *Clostridium* spp. is an anaerobic, Gram-positive, rod-shaped, and spore-forming bacteria found in the gastrointestinal tract, female genital tract, and less frequently on the skin [6,7]. Some species produce spores only under extremely unfavorable conditions and often appear as Gram-variable or Gram-negative [8].

In this report, we present the case of a *Clostridium symbiosum* (*C. symbiosum*) bacteremia in a 66-year-old woman. *C. symbiosum* was formerly known as *Fusobacterium symbiosum* because of its Gram-negative staining characteristics. Some anaerobes have an affinity to decolorize during Gram staining because of their specific sensitivity to the toxic effects of oxygen, which disrupts cell wall integrity [8]. *C. symbiosum *is a non-toxin-producing strict anaerobe with flagellum that may be found as a part of the human intestinal microbiota [9,10]. 

## Case presentation

A 66-year-old woman, with a history of bladder cancer and radical cystectomy with ureterosigmoidostomy nine years ago, was diagnosed with urosepsis in General Hospital Konjic. After nine days she was moved to the University Clinical Hospital Mostar because of worsening general condition. She was found to be soporous, dyspnoic, and hypotensive on admission. A multi-slice computed tomography (MSCT) of the brain was done and the result was normal. Laboratory investigation revealed an increased C-reactive protein (CRP) level, procalcitonin level, and leukocytosis. She also had anemia, hypoalbuminemia, and electrolyte imbalance (Table 1).

**Table 1:** Laboratory results - increased CRP and procalcitonin level, leukocytosis, anemia, hypoalbuminemia, and electrolyte imbalance CRP: C-reactive protein

Laboratory test	Result	Reference range	Unit
CRP	370	0.0-5.0	mg/L
Procalcitonin	12.42	<0.5 (low risk of sepsis) >2.0 (high risk of sepsis)	ng/mL
Leukocytes	11.5	3.4-9.7	x10^9^/L
Erythrocytes	3.03	3.86-5.08	x10^12^/L
Hemoglobin	88	119-157	g/L
Hematocrit	0.259	0.356-0.470	L/L
Albumin	23.1	40.6-51.4	g/L
Sodium	136	137-146	mmol/L
Potassium	5.4	3.9-5.1	mmol/L
Calcium	1.99	2.14-2.53	mmol/L

**Figure 1:**
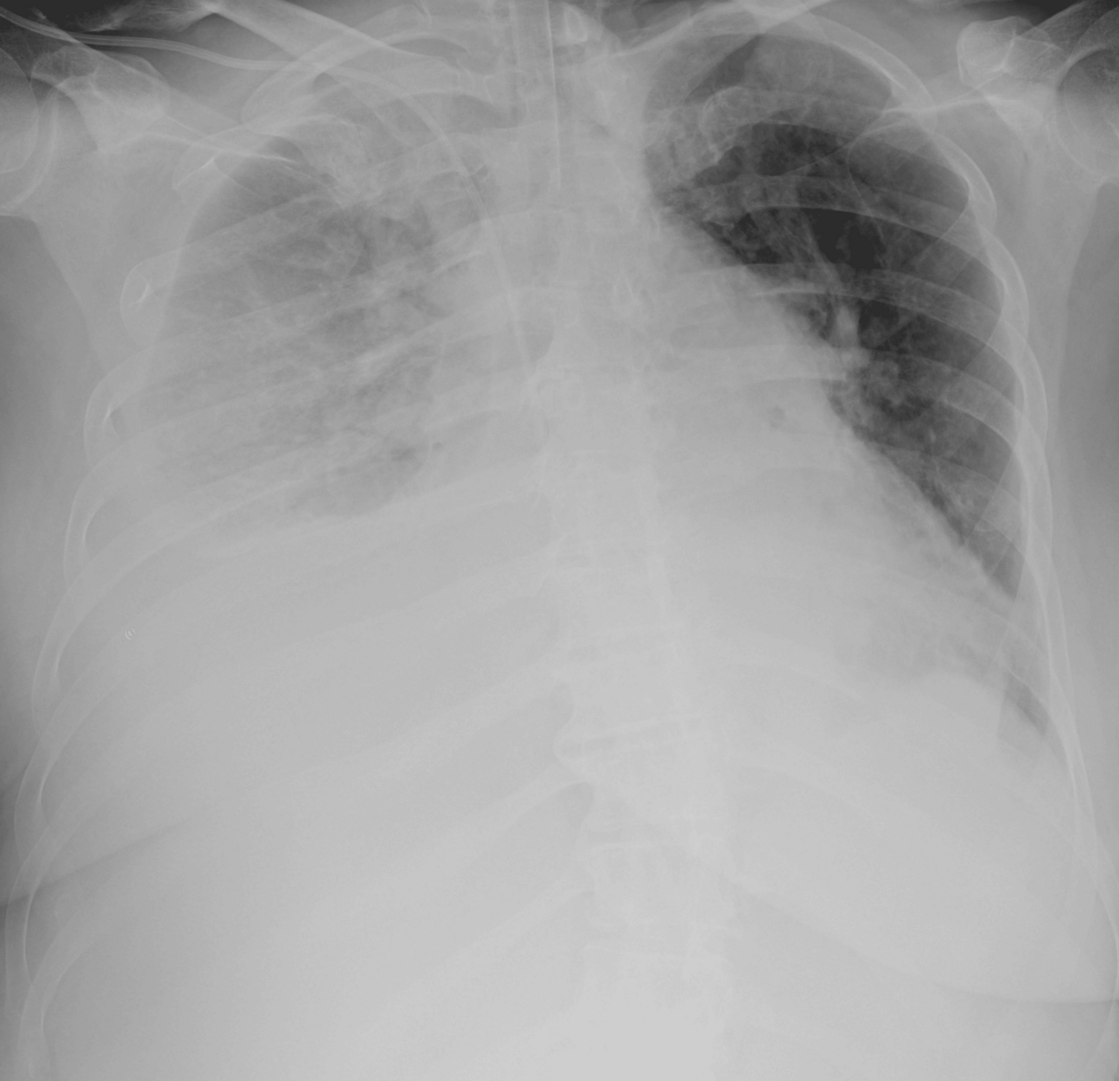
Chest X-ray - pleural effusion with confluent pulmonary infiltrates

One set of blood cultures was collected and sent to the microbiology laboratory. Intravenous empiric therapy including meropenem was immediately begun. The next day the patient´s general condition worsened again because of respiratory insufficiency. She was comatose (Glasgow Coma Scale 4), and dyspnoic. She was intubated and put on mechanical ventilation support with all necessary medications (antibiotic, probiotic, gastroprotective, anticoagulant, antiarrhytmic, corticosteroid and diuretic therapy, and vitamin K). Chest X-ray revealed pleural effusion with pulmonary infiltrates (Figure 1).

After 48 h of incubation in the BacT/Alert 3D system (bioMerieux Inc., Marcy-l'Étoile, France) the anaerobic bottle of the blood culture was positive. Gram stain revealed Gram-negative rod-shaped bacteria. There was no growth after subculturing to solid agar media (Columbia blood agar, Mast Group, Merseyside, UK) under aerobic conditions. After 48 h of incubation under anaerobic (GENbag anaer, bioMerieux Inc., France) conditions, there was a growth on Columbia blood agar - very small, grey, flat colonies. The isolate was identified by matrix-assisted laser desorption/ionization time-of-flight mass spectrometry (MALDI-TOF MS, Bruker, Ettlingen, Germany) as a *C. symbiosum*. Antibiotic susceptibility testing was performed using the Kirby-Bauer disk diffusion method on Columbia blood agar by EUCAST (European Committee on Antimicrobial Susceptibility Testing) standards [11]. The result showed susceptibility to meropenem, clindamycin, and metronidazole. In the next few days, the remaining two anaerobic bottles of the blood cultures that were sent to the microbiology laboratory during the hospitalization were positive and *C. symbiosum* was isolated.

The patient´s condition worsened - severe sepsis, hemodynamic instability, and respiratory insufficiency. Despite all appropriate measures hemodynamic collapse occurred, resulting in death 4 days after admission in University Clinical Hospital Mostar.

## Discussion

Only a few cases of human bacteremia caused by *C. symbiosum* are reported. The first case was reported by Elsayed and Zhang in a 70-year-old male patient with metastatic cancer of the colon [10]. Decousser et al. reported the second case in an immunocompetent 54-year-old man who went through sigmoidectomy and colorectal anastomosis because of diverticular sigmoiditis previously treated by antibiotic therapy [8]. Toprak et. al reported the third case in a 62-year-old woman with metastatic ovarian cancer who had cytoreductive surgery and partial sigmoid resection [12]. In 2023 Pan and Fan reported bacteremia caused by *C. symbiosum* in a two-year-old child who underwent partial colectomy after birth [13]. In the presented case, the patient had a history of bladder cancer that was treated with radical cystectomy and chemotherapy. All of these patients had a predisposition for clostridial bacteremia (CB). It is often associated with intraabdominal sepsis due to trauma or surgery, and malignancies or diabetes. Although CB is a rare clinical entity, it is reported that the mortality rate among patients with CB is as high as 50%. The presentation and outcome of CB depends on the type of *Clostridium* spp. causing infection and the host's underlying condition. Shah et al. reported that appropriate antibiotics should be initiated promptly in patients with a predisposition for CB. In their study mortality was notably lower in patients receiving appropriate antibiotics for *Clostridium* spp. [14]. In the presented case, the patient was admitted to a tertiary care center quite late with a presentation of severe sepsis, and that may be one of the causes of the death, despite an early recognition of CB and adequate therapy in the University Clinical Hospital Mostar.

Anaerobes are important pathogens in various human clinical infections. Most clinical microbiology laboratories perform limited anaerobic bacteriology and often no susceptibility testing at all due to high cost, technical skills, the requirement of prolonged incubation, and time delay in identification [15,16]. The application of 16S rRNA gene-sequencing-based identification and the MALDI-TOF MS have made it possible to provide rapid and reliable identification of numerous anaerobic species and timely information about their role in infections [17].

## Conclusions

In conclusion, *C. symbiosum* was a clinically significant isolate in this case because of the patient's clinical presentation and predisposition to clostridial infection, and the microbiological examination showed that three separate blood culture sets were positive for this microorganism. This is the first reported case of *C. symbiosum* bacteremia in Bosnia and Herzegovina. Early recognition and adequate therapy play a major role in preventing mortality and morbidity associated with anaerobic bacteremia. Incorporating new test methods into laboratory workflows may improve etiological diagnosis and facilitate the timely initiation of effective therapy for anaerobic bacteremia.
